# MODEM: multi-omics data envelopment and mining in maize

**DOI:** 10.1093/database/baw117

**Published:** 2016-08-08

**Authors:** Haijun Liu, Fan Wang, Yingjie Xiao, Zonglin Tian, Weiwei Wen, Xuehai Zhang, Xi Chen, Nannan Liu, Wenqiang Li, Lei Liu, Jie Liu, Jianbing Yan, Jianxiao Liu

**Affiliations:** ^1^National Key Laboratory of Crop Genetic Improvement, Huazhong Agricultural University, Wuhan 430070, China; ^2^College of Informatics, Huazhong Agricultural University, Wuhan 430070, China; ^3^School of Mechanical Engineering, Shandong University, Jinan 250061, China; ^4^School of Computer & software, Nanjing University of Information Science & Technology, Nanjing 210000, China

## Abstract

MODEM is a comprehensive database of maize multidimensional omics data, including genomic, transcriptomic, metabolic and phenotypic information from the cellular to individual plant level. This initial release contains approximately 1.06 M high quality SNPs for 508 diverse inbred lines obtained by combining variations from RNA sequencing on whole kernels (15 days after pollination) of 368 lines and a 50 K array for all 508 individuals. As all of these data were derived from the same diverse panel of lines, the database also allows various types of genetic mapping (including characterization of phenotypic QTLs, pQTLs; expression QTLs, eQTLs and metabolic QTLs, mQTLs). MODEM is thus designed to promote a better understanding of maize genetic architecture and deep functional annotation of the complex maize genome (and potentially those of other crop plants) and to explore the genotype–phenotype relationships and regulation of maize kernel development at multiple scales, which is also comprehensive for developing novel methods. MODEM is additionally designed to link with other databases to make full use of current resources, and it provides visualization tools for easy browsing. All of the original data and the related mapping results are freely available for easy query and download. This platform also provides helpful tools for general analyses and will be continually updated with additional materials, features and public data related to maize genetics or regulation as they become available.

**Database URL:** (http://modem.hzau.edu.cn)

## Introduction

Maize (*Zea mays* L.) is one of the most important crops worldwide. Also, as a model organism, maize promotes our understanding of plant genetics and genomes. With high-throughput techniques (including genotyping by array, RNA sequencing, metabolite identification and phenotypic measurement) maturing in recent years, there has been rapid growth in related maize multidimensional omics data (1–3). This large dataset will allow association mapping that will advance understanding of the genetic architecture of specific phenotypes, which, in turn, will allow deep annotation of the complex maize genome. While genetic and genomic research is progressing rapidly in maize, the related database resources have lagged behind and are limited for next level analyses—functional genome studies such as QTL mapping. Panzea (4), for example, contains genotype and morphological phenotype information from several populations. However, a multi-dimensional database including multiple levels of omics data, especially for the same panel, is urgently needed. Such a database should include abundant high-quality data on genomic variations, reliable quantitation of gene expression, detailed measurement of cellular metabolites, high-throughput characterization of morphological phenotypes for large, representative and diverse populations. In addition, mapping results based on these data and produced with different methods will directly contribute to further studies in the scientific community. The resource should also provide a user-friendly mechanism for retrieving data, convenient visualization tools and comprehensive annotation information.

We created the MODEM database to meet all these goals. Through wide collaboration, we have collected 527 maize elite inbred lines from which we have generated multiple omics-level datasets for examining variation or quantification of the genome, transcriptome, metabolism and phenome (Table 1). Importantly, we produced useful QTL mapping results based on these data with the linear mixed model. MODEM was developed to provide standardized data that can easily be retrieved and visualized, to better serve researchers.

**Table 1:** Multi-omics data involved in MODEM

Index	Data description
Germplasm resources	527 inbreds for association mapping panel (AMP) with different populations (143 lines for NSS, non-stiff-stock; 33 for SS, Stiff-stock; 232 for TST, Tropical and Semi-tropical; and the left 119 are regared as MIXED)
Genomic variation	∼50K SNPs from MaizeSNP50 BeadChip for AMP (513 lines), of which 368 have >1.03 million SNPs by RNA-Seq with 0.56 million passed the MAF > 0.05 filtering. The whole 513 panel is finally imputated to 0.56M SNPs
Transcriptome quantification	28 769^a^ genes’ quantitative expression of maize whole kernel (15 days after pollination, 15 DAP)
Phenotype measurement	nearly agronomic 50 traits including yield, response to drought, floods and diseases with 4–8 locations and multiple years (ranging from 2007 to 2012) of the whole AMP
Metabolomics	983 metabolic profiling of AMP and 17 amino acid components identified within 2 location for AMP
Mapping results	Mapping results of association analysis for different traits (including expression levels)

^a^Genes filtered as expressed in > 50% lines; there are 38 850 genes expressed in at least one line also could be obtained in DOWNLOAD page.

## Materials collection, data generation, processing and evaluation

### Plant germplasm

Currently, we have assembled a global germplasm collection with 527 elite inbred lines (association mapping panel, AMP) released from the major temperate and tropical/subtropical breeding programs of China, CIMMYT and the Germplasm Enhancement of Maize (GEM) project in the US, which were chosen to be representative of maize genetic diversity and/or for their promise in maize improvement. All of the lines were previously assayed by the 50K Maize SNP array (5, 6) (commercially available from Illumina). To further explore the genetic mechanisms controlling yield and to increase the yield of maize, deep RNA sequencing was also performed on 368 of the 527 lines using kernels harvested 15 days after pollination (DAP).

### Sampling and RNA library construction and sequencing

All 368 lines were planted in two replicate one-row plots in an incompletely randomized block design in Jingzhou, Hubei province of China in 2010 (2). Six to eight ears in each block were self-pollinated, and five immature seeds from three to four ears in each block were collected at 15 DAP. The collected immature seeds from the two replicates were bulked for total RNA extraction, and three additional lines (SK, Han21 and Ye478) were made for biological replicates. Total RNA was extracted using the Bioteke RNA Extraction kit (Bioteke, Beijing, China) according to the manufacturer’s protocol and cDNA libraries were constructed according to the manufacturer’s standard protocol (Illumina, Inc.). Libraries were amplified by 15 cycles of PCR with Phusion DNA polymerase (New England Biolabs, Inc.) and primers containing barcode sequences to distinguish different libraries during sequencing and data analysis. The average fragment size of each prepared library was 322 bp. Before loading libraries onto the flowcell, the libraries were quantified by qPCR, denatured with sodium hydroxide and diluted to 2.5 pM. Cluster formation, sequencing primer hybridization and 91 cycles of paired-end sequencing were carried out using reagents that Illumina supplied according to the standard protocol.

### Reads mapping, SNP calling and quality control

The 368 maize inbred lines were sequenced using 90-bp paired-end Illumina sequencing (raw RNA sequencing data have been deposited in NCBI Sequence Read Archive (SRA) under accession SRP026161). After trimming the adapter sequences and filtering out reads with low sequencing quality (>20 for base quality at leading and trailing ends, and at least 36 bp left in length), the RNA sequencing (RNA-seq) produced 70.1 million reads for each sample, totalling 25.8 billion high-quality reads. Short Oligonucleotide Alignment Program 2 (7) was used to map the reads against the B73 reference genome (AGPv2) with default parameters. Only reads that mapped uniquely to the genome were retained for further variation calling. On average, 71.0% of the reads were mapped to the B73 reference genome and 70.3% of the reads mapped to the maize annotated genes (filtered-gene set, release 5b). Among the genes with RNA-seq reads, 71.6% have coverage of >50% of the gene length. Of all the reads mapped to the genome, 83.5% were mapped uniquely and these reads were used to build the consensus sequence for each sample using SAMtools (8). Briefly, a two-step procedure was used to detect SNPs by carefully considering the characteristics of the RNA-seq data. In the first step, we identified the polymorphic loci in our population, and the population SNP-calling algorithm realSFS (9) was used to calculate the likelihood of variation for each covered nucleotide from the combined data of all of the 368 inbred lines. The variations with probability <0.99 or total depth <50 were filtered out. In the second step, we extracted consensus base, reference base, consensus quality, SNP quality and sequencing depth of each polymorphic locus for each inbred line using the Pileup command (8), and then considered the consensus base as the individual genotype with the following requirements: if the consensus base was different from the reference base, the non-reference allele must be the same as the non-reference allele detected from the population and the SNP quality must be ≥20. If the consensus base was the same as the reference base, the consensus quality must be equal to or >20 and the minimal depth must be ≥5. For sites that failed to pass these criteria, we regarded the consensus genotype as unreliable and assigned the individual genotype of those sites as missing. The 1 026 244 SNPs with missing rates <0.6 were then left to infer missing genotypes using fastPHASE (10). Heterozygous genotypes were masked as missing and all SNPs were named according to their physical positions in the reference genome (B73 AGPv2). Altogether, 558 629 SNPs with minor allele frequency (MAF) >5% remained for the subsequent QTL mapping analysis (the analysis using genomic variation for the purpose of finding which genes/regions associated with corresponding trait). The concordance rates were >99% between each pair of replicates and 98.6% when compared with overlapping genotypes determined by the MaizeSNP50 BeadChip. Among the 1 026 244 SNPs, 931 484 (90.8%) were mapped to within the 23 106 genes (filtered-gene set, release 5b). On average, there were 40.3 SNPs per gene. Whereas this SNP set includes 69.7% of SNPs reported in a previous study (11) on a nested association mapping population, it contains 7.5 times more exonic SNPs. This not only increases the probability that markers identified possess high linkage disequilibrium with target genes, but also helps in identification of causal variations. Finally, SNPs with a MAF < 5% were filtered out and the resulting 525 105 SNPs were then merged with 56 110 SNPs from the MaizeSNP50 BeadChip to produce the merged set of 558 650 SNPs, and the genotypes from Chip is preferred if inconsistent between the two sets.

### Gene expression profiles

To quantify the expression of known genes, reads that uniquely mapped to each gene within the reference genome (filtered-gene set, B73 AGPv2) were summed and normalized according to RPKM (reads per kilobase of exon model per million mapped reads). On average, there were 1540.7 reads for each whole gene for each individual. Genes having mapped sequencing reads (expressed) in more than half of the maize lines (28 769) were used for further eQTL mapping (the analysis for the purpose of finding which locus associated with gene’s expression variation). The expression values of each gene were then normalized using a normal quantile transformation to meet the assumption of detecting eQTLs through a linear mixed model that the expression values follow a normal distribution. This quantile transformation does not fully solve the problem, which only ensures that the phenotype is normal overall but not necessarily normal within each genotype class. However, it is a simple, sensible way to guard against strong departures from modelling assumptions with the small effect sizes typical in genetic association studies.

### Maize kernel metabolome profiling, identification and annotation

To extract metabolites of maize kernels, the association panel lines were planted in one-row plots in an incompletely randomized block design at three locations in China: Hainan (Sanya, E 109°51′, N 18°25′) in 2010 and Yunnan (Kunming, E 102°30′, N 24°25′) and Chongqing (E 106°50′, N 29°25′) in 2011 (3). All inbred lines were self-pollinated and ears of each plot were hand-harvested at their respective physiological maturity, followed by air drying and shelling. For each line, ears from five plants were harvested at the same maturity and 12-well growth kernels were randomly selected from five plants and bulked for grinding by using a mixer mill (MM 400, Retsch) with zirconia beads for 2.0 min at 30 Hz. The powder of each genotype was partitioned into two sample sets and stored at −80°C until extraction. One sample set was extracted for lipid-soluble metabolites, while the other was extracting for water-soluble metabolites. One hundred mg of powder and 1 ml absolute methanol, which contained 0.1 mg/l each of lincomycin and lidocaine, were used for lipid-soluble metabolites (or 70% methanol for water-soluble metabolites). Samples were extracted overnight at 4°C. After centrifugation at 10 000 g for 10 min, 0.4 ml of each extract was combined and filter spun using 0.22-μm filters (ANPEL, Shanghai, China, http://www.anpel.com.cn/) before analysis using an LC-ESI-MS/MS system. The metabolite quantification and annotation was performed by our newly developed method (12). To facilitate the identification/annotation of detected metabolites by our widely targeted metabolomics approach, accurate *m*/*z* of each Q1 was obtained, if possible. To this end, extracted ion chromatograms of the ESI-QqTOF-MS data for each of Q1 (*m*/*z* ± 0.2 Da) of the 983 transitions in the MS/MS library were manually evaluated for the presence of the target substances by analysing corresponding mass spectra, and accurate *m*/*z* values were obtained. For each of the corresponding accurate *m*/*z*, a fragmentation pattern was obtained by running the analysis under targeted MS/MS mode using three different collision energies of 10, 20 and 30 eV. The accurate m/z was assigned to the corresponding Q1 if similar fragmentation patterns were obtained between the ESI-Q TRAP-MS/MS and the ESI-QqTOF-MS/MS. Eventually, an accurate mass of 245 of Q1 was obtained. The MS/MS library was annotated based on the fragmentation pattern (delivered by ESI-Q TRAP-MS/MS and/or the accurate *m*/*z* value delivered by ESI-QqTOFMS/MS) and the retention time of each metabolite. Based on the annotation, commercially available standards were purchased and analysed using the same profiling procedure as the extracts. By comparing the *m*/*z* values, the retention time and the fragmentation patterns with the standards, 49 metabolites were identified, including amino acids, flavonoids and fatty acids (such as α-linolenic acid), and some phytohormones. For the metabolites that could not be identified by available standards, peaks in the MS/MS library, especially the peaks having similar fragmentation patterns with the metabolites identified by authentic standards, were used to query the MS/MS spectral data taken from the literature or to search the databases (MassBank, KNApSAcK, HMDB, MoTo DB and METLIN). Best matches were then searched in the Dictionary of Natural products and Kyoto Encyclopaedia of Genes and Genomes for possible structures. In all, 184 metabolites were identified and more than four different pathways were detected.

### Population structure, relatedness matrix and association analysis

A subset of 16 338 SNPs with <20% missing data and MAF > 5% were used to estimate population structure and kinship coefficients. STRUCTURE (13) was used to infer population structure with 10 000 replications for burn-in and MCMC processes, and five runs were performed at *k* = 3. The samples were divided into three subgroups, as previously suggested for this panel. The kinship matrix was calculated using the method introduced in Loiselle *et al*. (14). The associations between the extracted SNPs with MAF > 5% and all measured traits (including morphological phenotypes, kernel metabolome and transformed expression traits; Table 1) were analysed using the linear mixed model (LMM) (15) incorporating population structure and kinship using TASSEL (16). The significance cut off was set generally to 1/N, where N is the number of markers used, and all the results were provided to be displayed. The top six hidden confounding factors determined to be contributing to expression variability by Bayesian factor analysis (implemented in PEER) (17) were additionally included in the mixed model, in addition to population structure, to examine the validity of association significance for eQTL mapping.

## Database features and web interface

The Struts 2 framework (18) and B/S development pattern were used to develop MODEM. The back end of MODEM is implemented in JAVA and the web interface is implements using JSP, JavaScript, HTML5 Canvas and AJAX technologies. Apache Tomcat is used as the server to provide the webpage access service. These technologies allow the user to search and display their assignments conveniently by combining multiple types of data. All scripts involved in this study have been deposited into github (https://github.com/liujianxiao/MODEM).

### Genome browser for genotype and expression variation

**Figure 1.**
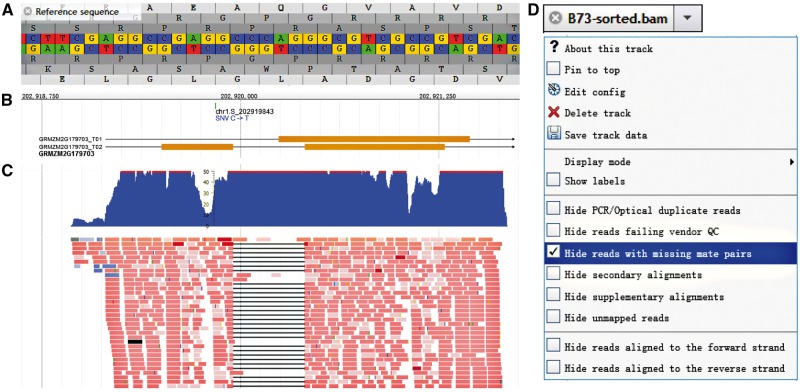
Genome Browser embedded in MODEM. (A) Sequence and six possible reading frames along the reference. (B) Gene and transcripts structure and SNP index, take GEMZM2G179703 as an example. (C) Original reads and coverage distribution of the gene referred above. (D) More options for each track in the drop-down menu, take BAM track as example.

JBrowse (19), built with JavaScript and HTML5, which is fast and embeddable, was used to build the genome browser of MODEM. The original sequencing reads and the qualitative and quantitative RNA-seq data were incorporated. Kinds of tracks (Table 2) could be selected to display. Reference sequence, with the translated amino acid sequence in six possible reading frames, can be displayed and are useful, especially when zooming in to the single gene level (Figure 1A). The structure and strand of both the gene and all transcripts can be viewed for the whole genome (Figure 1B), and specific information, including biotype, combined function annotation and sequences (for whole gene or each intron and exon) can be obtained by left-clicking on the gene body. The functional annotations were selected and combined from maizeGDB (20) and BioMart of Ensembl Plant (21). SNP attributes, such as referenced and variegated alleles, allele frequency and the potential functional consequences, especially of candidate eQTLs for other genes, can be viewed by clicking on the SNP ID after adding the SNP track. Further, users can obtain the original sequence and quality of each read after adding the BAM track and the coverage distribution along the chromosome with COVERAGE tracks (Figure 1C). MODEM also provides both density and X–Y plots to describe the expression quantitation in the QUANTITATIVE panel. In addition to being able to select or zoom in on a gene by right-clicking on the gene symbol, users can also choose the EXPRESSION COMPARISON option to compare all of the lines in a histogram, however, for those genes that were not expressed or did not meet our filtering criteria, a link can redirect the user to the Maize eFP Browser (22, 23) in maizeGDB to examine the expression pattern of these genes during the maize life cycle. JBrowse provides flexible sliding and scaling options, and for each track, more specific options can be selected, such as ‘Hide reads with missing mate pairs’ in the BAM track (Figure 1D).

**Table 2.** List and description of tracks in Genome Browser

Category name	Track(s) included	Description
Reference sequence	Reference sequence	Reference sequence and amino acids from six possible reading frames
Miscellaneous	GFF3	Gene structure annotation and expression comparison
SNP	SNPs from MaizeGo lab	SNP information including ID, effect type, eQTL information, allele frequency and alleles for each individual
Original reads	B73-sorted BAM	Sequences from original reads covered selected regions
Coverage	B73-sorted Coverage	Reads coverage for specific region in histogram
Quantitative Density/XYPlot	BigWig Density/XYPlot	Reads coverage for specific region in density plot or XYPlot

### Displaying specific information about materials/germplasms

Specific information, including the materials origins, population structure and pedigrees of the AMP can be searched, displayed and downloaded by the user. From this simple implementation, users can easily get a basic understanding and macro-impression of the germplasms.

### Search for genotypes by physical region or gene symbol

MODEM provides two sets of genotype data, the original and the filtered (by MAF < 5%) data sets. The former is primarily supplied for searching and genome browser functions, and the latter can be downloaded on the DOWNLOAD page. Information on SNPs can be queried by genomic region or gene symbol. For example, a region search with ‘chr1:12345.67890’ would display the SNPs between 12 345 bp and 67 890 bp on chromosome 1. The gene symbols should agree with common standards, such as GRMZM2G22222. SNPs from the RNA-seq are identified as ‘chr1.S_1000282’, where ‘chr’ indicates the chromosome, ‘S’ stands for SNP and 1000282 is the physical location of this variation. SNPs from the MaizeSNP50 BeadChip retain their original ID. The search results for the filtered genotype can be displayed in a table or saved as hapmap format.

### Displaying expression quantitation and eQTL results of whole maize kernels (15 DAP)

For the strict filtering of expressed genes, which were expressed in at least 50% of the lines, 28 769 genes were retained, and the list can be downloaded on the TRANSCRIPTOME page. The expression data is also available with RPKM normalization and further normal quantile transformation (Q–Q normed), and both can be searched and downloaded when a list of gene symbols provided. MODEM also provides a histogram in the genome browser for comparison of gene expression levels among individuals (described in detail above). Moreover, the RPKM-normalized data for the 38 769 genes that are expressed in at least one genotype are provided on the DOWNLOAD page. This data set may be more useful in some cases. It is also easy to search for eQTLs of interest on this page, and an approach for eQTL visualization is being explored.

### Search for phenotypes by trait name, location and year

In the MODEM interface, users can examine phenotypes of different populations or individuals by selecting trait name, germplasm of interest, corresponding year and/or location. The user can choose to display specific original phenotypes or use the selected results for BLUP calculation. The individuals of each phenotype are actual values (average 3–5 individuals/repeats), which may not show normal distribution. Details of the evaluation method of each phenotype and the working principles of our measurement system can be found on the MODEM webpage. Researchers can carry out genetic mapping themselves with downloaded phenotype data (including those with BLUP values), or they can search and download the pre-existing mapping results directly from the platform.

### Displaying association mapping results

Results for both association mapping and linkage mapping are obtained through selecting trait name, chromosome, year and location. Multiple traits can be simultaneously selected by holding down the CTRL key. MODEM provides intuitive visualization tools, zooming capabilities for much more detail and the ability to download publishable images in several familiar formats (PDF, PNG, JPG and SVG). In addition, the HIGHCHARTS technology (http://www.highcharts.com/) has been integrated to display the dynamic interactive JavaScript charts.

### Metabolomic data and related mapping results

**Figure 2.**
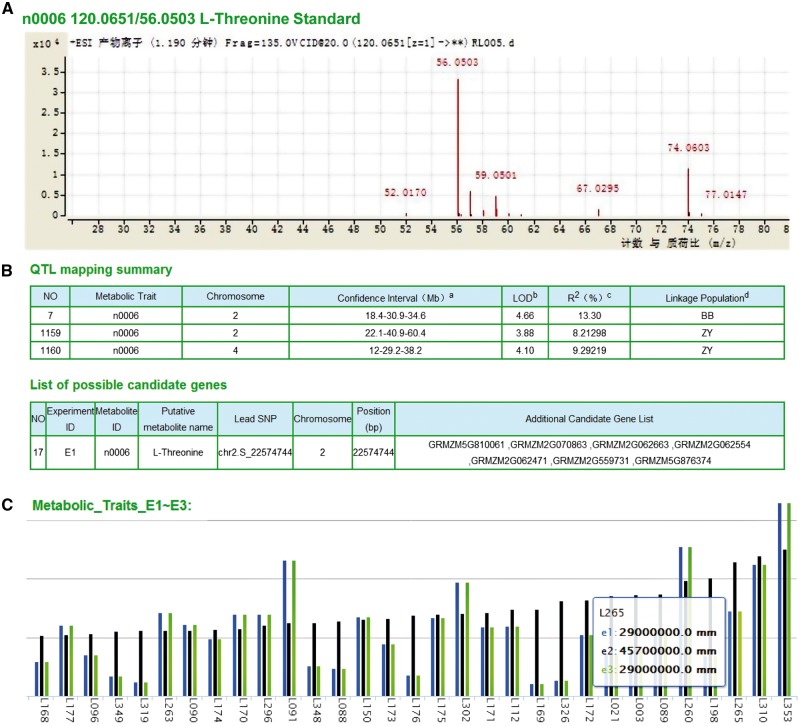
Original data and mapping results of metabolites. (A) Detailed spectrums features of L-Threonine (ID: n0006) based on LC-MS/MS analysis. (B) Significant QTL identified by linkage and association mapping and candidate genes of metabolite n0006. ^a^The number in the middle represents physical position of the peak marker, flanked by the left and right markers of the confidential interval of each QTL, respectively. ^b^LOD value for corresponding QTLs. ^c^The phenotypic variation explained by corresponding QTLs. ^d^BB and ZY correspond to linkage mapping conducted with B73/By804 and Zong3/Yu87-1 RIL populations, respectively. (C) Distribution of metabolite content within different experiments (E1∼E3: three biological repeats in three different locations) of each line (here represented anonymous by L+#ID), take AMP as example.

We have identified significantly metabolite-associated loci through metabolite-based genome-wide association studies (GWAS, to identify particular variants that are associated with the phenotype at the population level) and linkage mapping (to identify loci that cosegregate with the trait within families). Detailed metabolite information can be accessed and downloaded, including the list of 983 metabolite features (Figure 2A), the significant loci identified by GWAS across three environments (E1, E2 and E3), the linkage mapping summary based on the B73 × BY804 (BB) and ZONG3 × YU87-1 (ZY) recombinant inbred line (RIL) populations and the list of candidate genes (Figure 2B).

By either typing the compound name or selecting from the menu list, the features of LS-MS/MS identification (with pre-computed graphs) and the distribution in the AMP and the two RILs can also be shown in a barplot (Figure 2C).

### Additional tools assist further research

MODEM also provides some tools that may be needed in other types of analyses. For example, it is useful to know which variation within a particular region contributes to a known or even unknown genetype(s). To achieve this goal, an expedite tool called ‘T-test’ has been included. Users assign a region of interest and select a group of lines and hypothetical phenotypes. The tool divides the lines into subgroups based on the different genotype of each SNP, and a Student’s *t*-test is used to see if the selected phenotype shows significant differences between the subgroups. A matrix is generated that displays the *P*-value of each SNP on each phenotype, with significance indicating that the variation likely contributes to the specific trait. Additional tools, such as VENN plot capabilities, will be described on the website as they become available.

### Seed management

MODEM includes a seed information function, mainly to help with internal administration of seed storage, delivery and harvest. However, this information is also available to external researchers. Information about the seed species/line, source, and remaining quantity of each line available for loan can be retrieved and downloaded. For the administrator, they have more right on the management operations, such as importing, modifying or deleting seed information and the corresponding data. They also need to response to any requirement when received the email on the moment of submission finished. All the management operations can through single or bulk way and each record was kept and could be directly printed if linking to the printer.

### Download management

MODEM also includes a download management function that allows multidimensional omics data to be downloaded in the .csv, .txt and .xls formats. This aspect provides users with more information than is available on the SEARCH page.

## Conclusion and future work

MODEM integrates high-throughput maize multidimensional omics data, including information about genomic and transcriptomic variation, the metabolome of cellular traits and morphological phenotypes in a diverse sample set and provides related mapping results. It is a user-friendly, easily accessible, open interface with multiple functions to help researchers store, search and analyse data, thus promoting maize genetics research. The MODEM platform could also be modified to be applied to other systems or species.

This platform mainly focused on the functional genomics of maize and will continue to be optimized and, and in the future, more data and tools will be included and developed, including higher density variant map (for example by adding genotypes from 600K and GBS strategies; Liu *et al.*, in revision) and diverse variation (such as structural variation and presence and absence variation of TE), more phenotypes and related mapping results (like disease-related and drought-related traits) (24, 25), tool for primer design by considering diverse variant and interfaces to R (https://www.r-project.org/) to rapid construct haplotypes and gene regulatory network. We also will integrate other public data sets focused on the reference genome, such as expression pattern along different tissues also measured by RNA-seq (26), mapping results (especially GWAS) from other groups, and epigenetic modifications including differentially methylated regions (DMRs) (27) or even MNase-hypersensitive regions (means open chromatin) (28) to together promote maize genetic studies.

## Funding

National Key Research and Development Program of China (2016YFD0101001), National Natural Science Foundation of China (31123009, 31222041), National Basic Research and Development Program of China (2011CB100105), National Hi-Tech Research and Development Program of China (2012AA10A307).

*Conflict of interest*. None declared.
